# Antineoplastic effect of iodine in mammary cancer: participation of 6-iodolactone (6-IL) and peroxisome proliferator-activated receptors (PPAR)

**DOI:** 10.1186/1476-4598-8-33

**Published:** 2009-06-06

**Authors:** Carmen Aceves, Pablo García-Solís, Omar Arroyo-Helguera, Laura Vega-Riveroll, Guadalupe Delgado, Brenda Anguiano

**Affiliations:** 1 Instituto de Neurobiología, Universidad Nacional Autónoma de México, Campus UNAM-Juriquilla, Querétaro, 76230, México

## Abstract

**Introduction:**

Studies in mammary cancer demonstrated that moderately high concentrations of molecular iodine (I_2_) have a antiproliferative and apoptotic effect either *in vivo *as *in vitro*, however the cellular intermediated involved in these effects has not been elucidated.

**Methods:**

Virgin Sprague-Dawley rats were treated with methyl-nitrosourea (MNU: single dose ip, 50 mg/Kg bw) and the participation of arachidonic acid (AA) and PPAR receptors in the antineoplasic effect of I_2 _where analyzed.

**Results:**

I_2_-treated rats for four weeks exhibited a significant reduction in the incidence (62.5 vs. 100%) and size (0.87 ± 0.98 vs 1.96 ± 1.5 cm^3^) of mammary tumors. HPLC analysis showed that tumoral but not normal mammary tissue contained an elevated basal concentration of AA and significantly more AA-iodinated called 6-iodolactone (6-IL) after chronic I_2 _treatment. Tumors from I_2_-treated rats showed fewer cells positive to proliferating cell nuclear antigen, lower blood vessel density, as well as decreases in vascular endothelial growth factor, urokinase-type plasminogen activator, and PPAR type alpha (PPARα). These same tumors showed increases in the cell death markers, TUNEL-positive cells (p < 0.05) and the enzyme caspase-3 (trend), as well as significant induction of PPAR type gamma (PPARγ).

**Conclusion:**

Together, these data demonstrate that the antineoplasic effect of iodine involves 6-IL formation and PPARγ induction.

## Introduction

In recent years, it has become evident that molecular iodine (I_2_) participates in the physiology and/or pathology of extrathyroidal organs like mammary gland, stomach, prostate, etc. [1-3]. In mammary gland I_2 _but not iodide (I^-^) supplementation alleviates human mastalgia and exerts a potent antineoplasic effect on pharmaco-induced mammary tumoral progression in rats [4-7]. In thyroid gland, I^- ^has an apoptotic effect that is mediated by derivatives of arachidonic acid (AA) or eicosapentaenoic acid such as 6-iodo-5-hydroxy-8,11,14-eicosatrienoic acid, also called 6-iodolactone (6-IL), or alpha-iodohexadecanal, respectively [8-10]. The formation of these iodolipids requires, in addition to iodide uptake by specific transporters, its oxidation by thyroperoxidase (TPO). The oxidized iodine intermediate has not been identified, but one of the candidates is I_2 _[11,12]. We reported that mammary glands of virgin rats and MNU-induced tumors take up ^125^I_2 _by mechanisms independent of the sodium iodine symporter (NIS) or pendrin (PEN) [13]. These findings were corroborated in the human tumoral cell line MCF-7, where we found that ^125^I_2 _is taken up by a facilitated diffusion system and covalently bound to lipids similar to 6-IL in both the presence or absence of peroxidase inhibitors, indicating that the oxidized iodine form I_2 _is organified in the absence of peroxidases [14]. Our data also shows that cancerous cells are more sensitive than the normal mammary cells to the apoptotic effect of I_2_, but they are equally sensitive to 6-IL; this suggests the presence of abundant 6-IL precursor in tumoral cells [15]. 6-IL is formed by the iodination of AA, which is an essential membrane lipid, and elevated AA has been widely implicated in tumoral process [16,17]. Although the cellular mechanism involved in the 6-IL apoptotic effect has not yet been elucidated, we have proposed that the peroxisome proliferator-activated receptors (PPAR) could be the mediator [3]. These ligand-dependent transcription factors are involved in regulating cell cycle control and apoptosis of several cancer cells [18,19]. Recently, our laboratory showed that 6-IL is a specific ligand of PPARs with almost 6-fold higher affinity than AA, which is one of the endogenous ligands of these receptors [20]. In the present work we analysed, in an *in vivo *model, the presence of AA and formation of its 6-IL derivative in normal and tumoral mammary gland after chronic supplement of I_2_. We also characterized the cellular pathways involved in the antineoplasic effect of I_2 _treatment.

Our data show that tumoral tissue contains 5-fold more AA and produce 12 times more 6-IL than normal mammary tissue, and that this increase in 6-IL is accompanied by decreases in invasive signals as well as increases in apoptosis indicators. In normal mammary gland from I_2_-treated rats, these parameters have not shown any changes compared to untreated rats. Together, these data demonstrated that the antineoplasic effect of iodine involves the formation of 6-IL and PPARγ induction.

## Materials and methods

### Animals

Virgin, female Sprague-Dawley rats, 5 weeks of age, were obtained from the vivarium of the Instituto de Neurobiología, UNAM-Juriquilla. Rats were housed in a temperature-controlled room (21 ± 1°C) with a 12-h light/dark schedule. They were provided with food (Purina rat chow; Ralston Purina Co., St. Louis, Missouri, USA) and water *ad libitum*. All of the procedures followed UNAM Animal Care and Use Committee guidelines.

### Iodine treatment and carcinogenesis induction

At 5 weeks old, rats were sorted into 2 experimental groups using a randomization process, and iodine treatment was started. The experimental groups were: a) Control and b) 0.05% I_2 _in drinking water (I_2_). Distilled water was used for drinking water and for all solutions. A saturating solution of I_2 _(1.33 × 10^-3 ^M) was prepared, and the concentration of 0.05% was confirmed by titration with sodium thiosulfate (Kenkel, 1994). Treatments were continued until 16 weeks after carcinogen injection. Two weeks after the I_2 _treatment began, subgroups of rats (control and I_2 _treatment) were anesthetized with a mixture of ketamine and xylazine (Cheminova, México, 30 mg and 6 mg, respectively, per Kg bw) and given a single intraperitoneal injection of 50 mg of MNU (Sigma, St. Louis, Missouri, USA) per Kg bw. MNU was dissolved and activated by heating to 50–60°C in 0.9% saline, pH 5.0 [21].

### Evaluation of mammary gland carcinogenesis

Rats were weighed and palpated for tumors every week beginning 4 weeks after and ending 16 weeks after MNU administration. A tumor was defined as a discrete, palpable mass recorded for at least 2 consecutive weeks. Tumor incidence was calculated as the percentage of animals with one or more palpable tumors. Tumor multiplicity was calculated as the average number of tumors per animal in each treatment group. The mean latency of tumor onset for each treatment group was calculated as the mean time interval (in weeks) from MNU injection to the appearance of the first palpable tumor. At the end of the experiment the rats from each group were sacrificed by decapitation. Tumor sizes were measured using a caliper, and the volumes were calculated by the ellipsoid formula [21]. If the tumors reached the ethically maximal size of 3 cm^3 ^(1.5 × 2.0 cm), rats were anesthetized with the ketamine/xylazine mixture, and the tumors were surgically removed even before the end of the experiment. Normal or tumoral mammary glands were fixed in 10% neutral buffered formalin or frozen in dry ice and kept at -70°C. Blood was collected to determine T3 circulating levels.

### T3 circulating levels

Serum T3 levels were measured by the homologous RIA method previously described [7].

### Histopathological analysis

Fixed mammary tumors were embedded in paraffin blocks. Sections of 5-μm thickness were cut from each block and placed on slides that had been treated with 3-aminopropyltriethoxysilane. Sections were deparaffinized in xylene, rehydrated in descending grades of ethanol, and stained with hematoxylin and eosin. Tumors were classified according to criteria published by Russo & Russo [22].

### Arachidonic acid (AA) and 6-iodolactone (6-IL) identification

Normal and tumoral mammary tissues were collected and stored frozen; 500 mg tissue samples were used for neutral lipid extraction according to the procedure described by Boeynaems and Hubbard (1980), and the extracts were evaporated to dryness under a stream of N_2 _and stored at -80°C. Before HPLC (BIO-RAD 2800, Hercules California, USA) analysis, the neutral lipids were dissolved in 100% EtOH, and a standard curve was constructed with 10–500 nmol of pure AA (Amershan, Arlington Heights, Illinois, USA) or 6-IL dissolved in ethanol. The samples (100 μl) were injected into the μBondapak C_18 _column (3.9 × 300 mm; particle size, 10 μm; WATERS, Milford, Mississippi, USA) maintained at room temperature. Elution was performed with a mobile phase gradient using solution A (CH_3_CN-H_2_O; 1:2, vol/vol) and solution B (CH_3_CN-H_2_O; 2:1, vol/vol) at 1 ml/min. Absorption was measured at 206 nm.

### Immunohistochemistry of proliferating cell nuclear antigen (PCNA)

Five-μm sections of mammary tumors from rats with or without I_2 _treatment were deparaffinized, rehydrated, and subjected to antigen retrieval (10 mM sodium citrate) at 80°C for 20 minutes. After cooling at room temperature, sections were treated with 0.3% hydrogen peroxide to block endogenous peroxidase activity. Non-specific binding was blocked with 2% dried fat-free milk in 20% fetal bovine serum-PBS solution (1 hr at 37°C). Sections were incubated at room temperature for 30 min in a humid chamber with mouse monoclonal anti-rat PCNA, clone PC10 (DakoCytomation, Carpinteria, California, USA), diluted 1:150. Immunocomplexes were visualized by goat anti-mouse-immunoglobulin, peroxidase labeled (EnVision™+System, peroxidase, DakoCytomation, Carpinteria, California, USA). Diaminobenzidine (DAB) was used as the chromogen to generate a brown precipitate after reaction with peroxidase. Sections were counterstained with hematoxylin, rinsed, dehydrated, and mounted with Entellan (Merk, Darmstadt, Germany). Cells expressing the antigen were identified by a brown stain over the nucleus. Tumor sections were incubated without either the primary or secondary antibody to test for the specificity of the antibody. PCNA-positive cells were identified by a brown stain over the nucleus. Labeling indices were obtained by counting the number of labeled cells among at least 500 cells per region, and 5 random regions were analyzed.

### Quantification of blood vessels

The protocol described was used to prepare the sections for immunofluorescence. Blood vessels were marked using the anti-mouse antibody coupled to fluorescein isothiocyanate (Alexa Fluor 488; Molecular Probes, Eugene, OR). This antibody stained blood vessels in all samples, as confirmed by dual label immunofluorescence studies with anti-PECAM-1 [23], and the number of stained blood vessels was determined visually (5 areas per slide) with a 10× objective. Images were digitized and evaluated with image-analysis software. Blood vessels were outlined, and their areas were calculated.

### Quantification of in situ cell death

Cell death was detected in formalin-fixed, paraffin-embedded tumor sections using the *in situ *cell death detection kit, fluorescein, POD (Roche Molecular Biochemicals, Mannheim, Germany), which is based on the terminal deoxynucleotidyl transferase-mediated dUTP nick end labeling (TUNEL) method. Sections (5 μm) were prepared and treated according to the manufacturer's instructions. DAB was used as the chromogen, and sections were counterstained with hematoxylin. TUNEL-positive cells were identified by a brown stain over the nucleus. Five regions, chosen at random, were analyzed, and labeling indices were obtained by counting the number of labeled cells among at least 500 cells per region and expressed as percentage values.

### Caspase-3 activity

Caspase-3 activity was measured using a standard colorimetric kit (Sigma, St. Louis, MO). Briefly, frozen mammary tumors were homogenized (1:2) with lysis buffer (50 mM HEPES, pH 7.4, 5 mM CHAPS, 5 mM DTT, 4°C). Samples were centrifuged at 16, 000 *g *for 20 min, 4°C, and supernatants were collected and stored at -70°C. Protein concentrations were determined by the Bradford method (BIO-RAD protein assay, Richmond, CA), and 100 μg of protein were assayed for caspase activity. The substrate was 200 μM DEVD-pNa, which contains the chromophore *p*-nitroanilide (pNa) linked to a synthetic tetrapeptide DEVD (acetyl-Asp-Glu-Val-Asp). Final cocktail (300 uL) were incubated in assay buffer (20 mM HEPES, pH 7.4, 2 mM EDTA, 0.01% CHAPS, 5 mM DTT) for 3 h at 37°C with continuous agitation and protected from light in a 96-well plate. Samples were read at 405 nm in an ELISA reader, and caspase activity was expressed as nmol of pNa released/h/mg protein.

### Expression of vascular endothelial growth factor (VEGF), urokinase-type plasminogen activator (uPA), and PPAR receptors

VEGF, uPA, and the alpha and gamma PPAR receptor isoforms were quantified by quantitative real-time PCR (qPCR). Total RNA from normal and tumoral mammary gland from rats with or without I_2 _treatment was extracted using TRIzol reagent (Life technologies, Inc) according to the manufacturer's instructions. Two micrograms of total RNA was reversed transcribed using the Superscript II system (Invitrogen, Carlsbad, CA). PCR was performed on the sequence detector system Roto-Gene 3000 (Corbett Research, Mortlake, NSW) using SYBR green as a marker for DNA amplification. The reaction was performed with 1 μL cDNA template and the qPCR supermix-UDG kit (Invitrogen), using 40 cycles of three-step amplification (94°C for 30 s, 55–58°C for 30 s, 72°C for 30 s) and the gene-specific primers listed in Table 1. PCR generated only the expected specific amplicon, which was demonstrated in each case by the melting temperature profile (dissociation curve) and by electrophoresis of 5 μL of the PCR product through a 2% agarose gel containing ethidium bromide in TAE buffer. No PCR products were observed in the absence of template. Gene expression was calculated using the Dcycle threshold (Dct) method and normalized to the content of Cyclophilin (Cyc), a non-regulated housekeeping gene [13]. All measurements were performed in triplicate.

**Table 1:** Oligonucleotides

PPAR-α	M88529	tgtatgaagccatcttcacg	ggcattgaacttcatagcga	163
PPAR-γ	AF156665	tcaaaccctttaccacggtt	caggctctactttgatcgca	147
β-actin	NM-031144	gtcccagtatgcctctggtcgtac	ccacgctcggtcaggatcttcatg	171
VEGF	Nakamura et al, 1996	gctctcttgggtgcactgga	caccgccttggcttgtcaca	563 431
uPA		gtggccaaaagactctgagg	caagcgtgtcagcgctgtag	267

### Statistical analysis

The effects of dietary treatments on mammary cancer incidence were analyzed using 2 × 2 contingency tables and a one-tail chi-square with Yates' correction test. The effects of treatments on tumor multiplicity, tumor latency, tumor size, caspase-3 activity, PCNA, TUNEL, and vascular area counts were analyzed using the unpaired t test; for the other variables, two-way ANOVA analysis was performed to determine the significance of differences between groups, followed by Tukey's test for the significance of differences among multiple experimental groups. Values with p < 0.05 were considered statistically significant.

## Results

Mammary glands were evaluated both macro- and microscopically for the presence of tumors. Various combinations of papillary, cribriform, or comedo mammary carcinomas were detected, and no correlation between histological type of mammary cancer and treatment was observed (data not shown). The effect of long-term I_2 _treatment on mammary carcinogenesis is summarized in Table 2. Cancer incidence was 37.5% lower in I_2_-treated than in control rats, whereas the number of tumors per rat and latency were similar for all groups. As shown in the table 3, body weight and thyroid status remained similar in all groups.

**Table 2:** Effect of long-term I_2_-treatment on mammary tumorigenesis after 16 weeks of MNU administration.

Treatment	No. of rats with tumors	%	Tumor latency (weeks)^†^	Tumors per rat^†^
MNU	10/10	100	11.9 ± 2.8	2.5 ± 1.7
I_2_-MNU	10/16*	62.5*	11.6 ± 1.4	2.0 ± 0.9

At 5 weeks of age, rats were treated with 0.05% I_2 _in the drinking water. Two weeks later, rats received a single i.p. injection of MNU (50 mg/Kg bw), and the administration of I_2 _was continued until 16 weeks had elapsed. ^† ^indicates mean ± SD. Asterisk (*) indicates P < 0.05 compared to MNU.

**Table 3:** Body weight gain and T3 levels after 16 weeks of iodine treatment.

Treatment	Body weight gain (g)	T3 (ng/dl)
Control	154 ± 15	63.7 ± 10
MNU	157 ± 12	67.9 ± 3

Control + I_2_	152 ± 9	63.2 ± 11

MNU + I_2_	150 ± 16	60.2 ± 5.6

**Figure 1:**
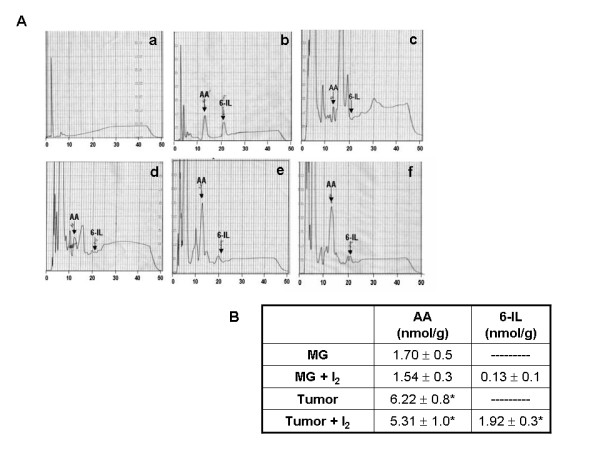
**Chromatographic analyzes**. Quantification by HPLC of arachidonic acid (AA) content and 6-iodolactone (6-IL) formation in normal (MG) or tumoral (Tumor) mammary glands from rats with or without molecular iodine supplements. A, representative chromatograms; a) base line, b) standards, c) MG control, d) MG +I_2_, e) tumor, f) tumor + I_2_. B, quantification; data are expressed as the mean ± SD (n = 10). Differences between experimental groups were analyzed using a one-way ANOVA and the Tukey-HSD Test. *indicates significant difference from the appropriate control (p < 0.05)

Our first objective was to determine the concentrations of AA and 6-IL in normal and tumoral mammary tissue with and without I_2 _treatment. As shown in the figure 1, tumoral tissue contains 5-fold more AA than normal mammary gland, and after the I_2 _treatment, both normal and tumoral tissue contain detectable 6-IL; however, the concentration in tumoral tissue is 12 times greater than in the control.

**Figure 2:**
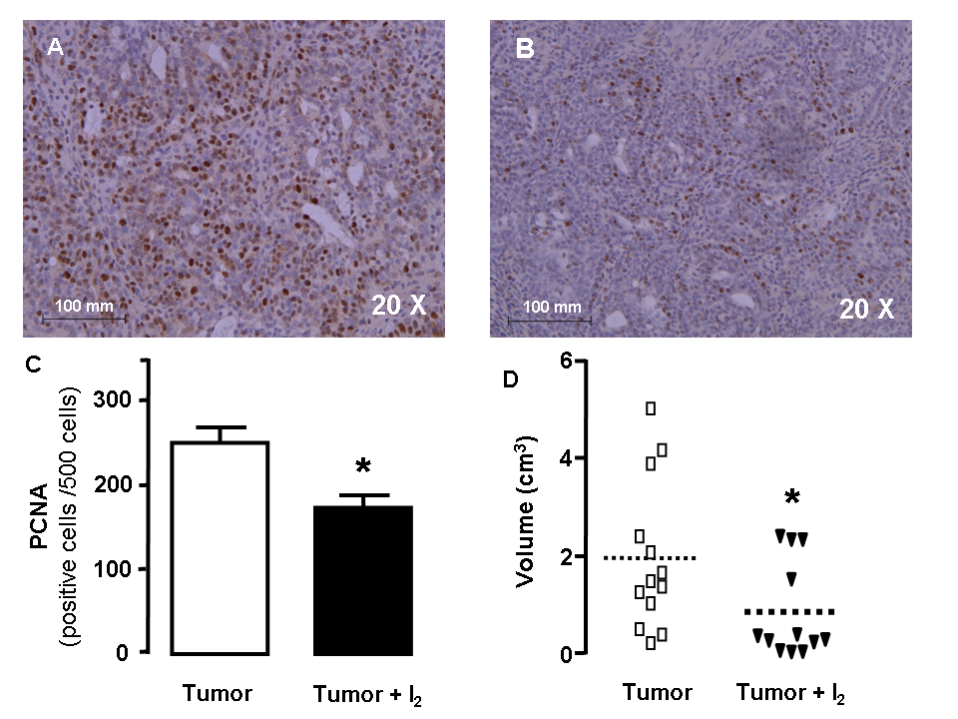
**Proliferative rate**. Immunohistochemical presence of PCNA-positive cells in tumors from control (A) and I_2_-supplemented rats (B); PCNA-positive cells were revealed with diaminobenzidine (brown stain) and counterstained with hematoxylin (purple stain). C, quantification; D, size of tumors (Volume). Differences between experimental groups were analyzed using an unpaired t test. *indicates significant difference from the appropriate control (p < 0.05).

Figure 2 shows the analysis for cell proliferation in mammary tumors of control and I_2_-treated rats. Tumors of control rats had significantly more PCNA-positive epithelial cells than tumors of I_2_-treated rats (250 ± 51 vs 173 ± 46 positive cells/500 cells) p < 0.05%. Moreover, mammary tumors of I_2_-treated rats were smaller than those of controls (0.87 ± 0.98 vs. 1.96 ± 1.5 cm^3^, p < 0.05%).

**Figure 3:**
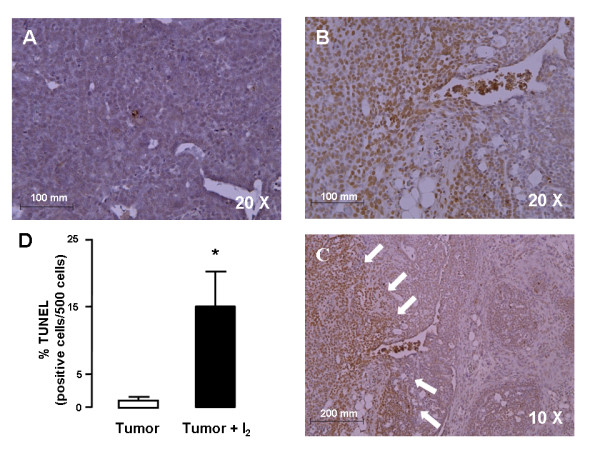
**Apoptotic rate**. Representative sections of TUNEL-positive cells in tumors from control (A) or I_2_-treated rats (B), magnification 20×. C, Section of rat tumor treated with I_2 _that shows an extensive zone of TUNEL-positive cells (white arrows), magnification 10×. TUNEL-positive cells were revealed with diaminobenzidine (brown stain) and counterstained with hematoxylin (purple stain). D, TUNEL-positive cell quantification. The difference between experimental groups was analyzed using an unpaired t test. *indicates significant difference from the control (p < 0.05)

To evaluate the effect of I_2 _treatment on cell death, we used the TUNEL method. Figure 3 shows representative tumor sections of control and I_2_-treated tumors. Interestingly, mammary tumors of I_2_-treated rats showed specific zones with numerous TUNEL-positive cells, whereas control tumors have few and disperse TUNEL-positive cells. The mammary tumors of I_2_-treated rats had a much higher percentage of TUNEL-positive cells (15 ± 5.5%) than the control tumors (1.0 ± 1.1%).

**Figure 4:**
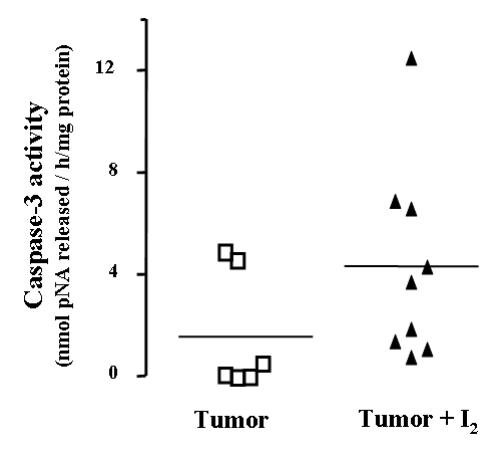
**Caspase-3 activity**. Individual tumors from control or I_2_-treated rats were assayed; horizontal line represents the mean of each group. Differences between experimental groups were analyzed using an unpaired t test. A tendency toward higher caspase-3 activity (p = 0.246) in I_2_-treated tumors was observed.

To clarify if the cell death induced by I_2 _is apoptosis, we measured the activity of the enzyme caspase-3 (figure 4). We found a tendency toward higher caspase-3 activity (p = 0.246) in I_2_-treated tumors than in control tumors.

**Figure 5:**
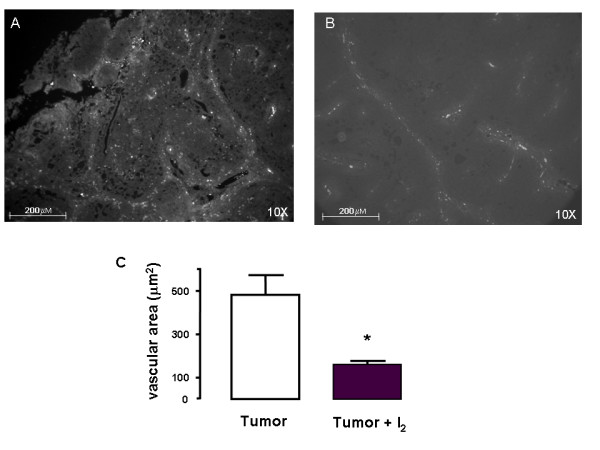
**Vascular area**. Immunofluorescence blood vessels in tumors from control (A) and I_2_-supplemented rats (B). Vessel area was calculated as the total field area positively stained for the vascular marker (μm^2^). C, quantification. Differences between experimental groups were analyzed using an unpaired t test. *indicates significant difference from the control (p < 0.05)

**Figure 6:**
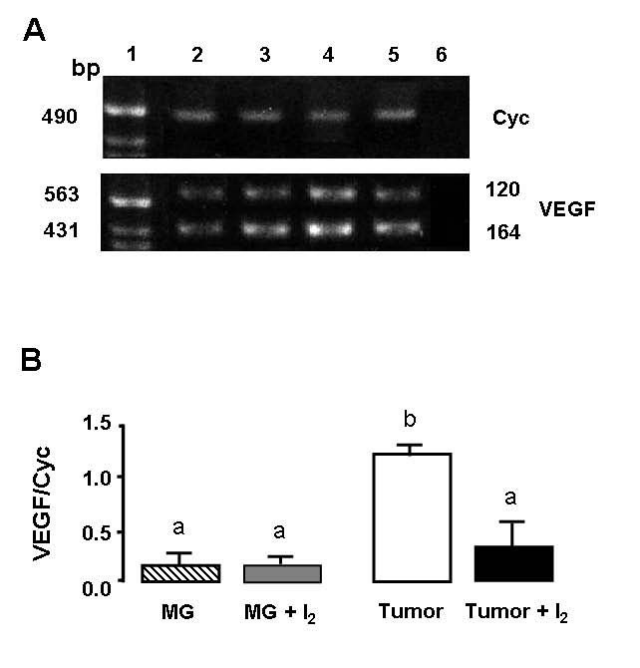
**Vascular Endothelial Growth Factor (VEGF) expression**. Isoforms of VEGF mRNA in normal (MG) or tumoral (tumor) mammary glands from control or I_2_-treated rats were measured by the real time PCR method. A. Representative gel of amplicon of Cyclophilin (Cyc) and 120 and 164 isoforms of VEGF mRNA in 2% agarose gel stained with ethidium bromide. 1, ladder; 2, MG; 3, MG + I_2_., 4, Tumor; 5, Tumor + I_2_; 6, all the PCR reagents without RT sample. B. VEGF mRNA quantification. Cyclophilin (Cyc) served as internal control and was used to normalize for differences in input RNA. Data are expressed as the mean ± SD. Differences between experimental groups were analyzed using a one-way ANOVA and the Tukey-HSD Test. Means with different letters are significantly different (p < 0.05)

**Figure 7:**
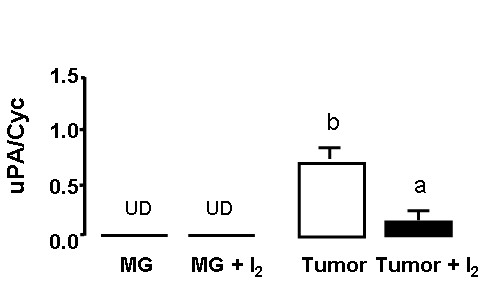
**Urokinase Plasminogen Activator (uPA) expression**. uPA mRNA in normal (MG) or tumoral (tumor) mammary glands from control or I_2_-treated rats were measured by the real time PCR method, and Cyclophilin (Cyc) served as internal control. UD, undetectable levels. Data are expressed as the mean ± SD. Differences between experimental groups were analyzed using a one-way ANOVA and the Tukey-HSD Test. Means with different letters are significantly different (p < 0.05)

To evaluate the effect of iodine on tumoral angiogenesis and invasive potential, we analysed the vasculature area and the expression of VEGF, as well the expression of uPA, respectively. Figures 5, 6, and 7 show that tumors from I_2_-treated rats exhibited significant decreases in vasculature area as well as lower expression of VEGF and uPA mRNA. They also show that in normal mammary tissue the I_2 _treatment does not induce any changes in VEGF expression (figure 6) and that uPA expression remains undetectable (figure 7).

**Figure 8:**
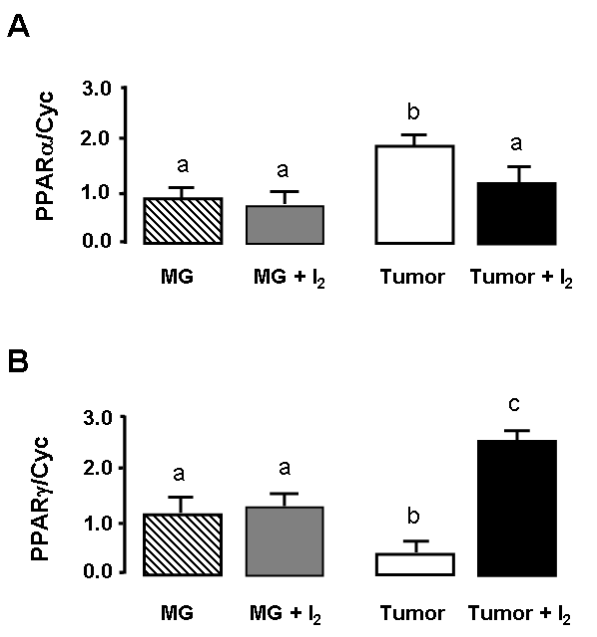
**PPAR expression**. Isoforms of PPAR mRNA in normal (MG) or tumoral (tumor) mammary glands from control or I_2_-treated rats were measured by the real time PCR method. A, PPAR type alpha (PPARα). B, PPAR type gamma (PPARγ). Cyclophilin (Cyc) served as internal control and was used to normalize for differences in input RNA. Data are expressed as the mean ± SD. Differences between experimental groups were analyzed using a one-way ANOVA and the Tukey-HSD Test. Means with different letters are significantly different (p < 0.05)

The last objective of this study was to analyze the participation of PPAR receptors in the antineoplastic effect of iodine. Figure 8 shows in first time that untreated mammary tumors express significantly higher levels of PPARα and significantly lower levels of PPARγ than normal mammary gland. The second find is that I_2 _treatment exerts a significant decrease in the expression of PPARα and a significant increase of the PPARγ isoform in comparison with control tumors. In normal mammary gland; I_2 _treatment does not modify the expression of either of these receptors.

## Discussion

This report confirms our previous observations that I_2 _treatment reduces mammary cancer incidence [7], decreases the proliferative rate (PCNA), and induces apoptosis (TUNEL and caspases) in cancerous mammary cells in vitro [14,15] or in vivo without any secondary adverse effect on the thyroid or general health [13]. One interesting effect of I_2 _treatment observed in these tumors was the generalized reduction in proliferation and vasculature, but the regionalized zones with positive apoptotic signaling (TUNEL) suggest the existence of cell populations with differential I_2_-sensitivity Although we cannot yet explain this pattern, it is well known that tumors are heterogeneous populations of cells. Studies in our laboratory [15] have shown that cancer cells – either differentiated or not – are more susceptible to the apoptotic effect of I_2 _than the corresponding normal mammary epithelium (MCF-7 > MDA-MB231 > MCF-12F cells lines, respectively). Indeed, this characteristic pattern of apoptotic zones could account for the lack of significant differences in caspase-3 activity, since it was measured in homogenized pieces of tumors; it may also account for our previous observation that tumor growth resumes if I_2 _treatment is suspended. [7]. The specific components involved in this I_2_-sensitivity are not known; however the content of lipids that can be iodinated is an attractive possibility.

The present work demonstrates that tumoral tissue contains significantly higher concentrations of arachidonic acid than normal mammary tissue and that the I_2 _treatment is accompanied by a 12-fold increase in 6-IL formation. These findings support our earlier hypothesis that the antiproliferative effect of I_2 _is mediated by the formation of the 6-IL [3]. In thyroid, the apoptotic effect of iodine excess is mediated by 6-IL and/or by iodohexadecanal, both of which are generated by the oxidation and organification of I^- ^by TPO [9,10]. In this regard, we reported that in thyroid, excess I_2 _exerts, as I^-^, an inhibitory effect on NIS, PEN, TPO, and deiodinase type 1 expression, but without the transient decline in circulating thyroid hormone (TH) levels. This finding indicates that I_2 _can bind to organic components and thereby inhibit gene expression and also generate TH. Moreover, the finding that I_2_-treated animals supplemented with methimazole preserve the thyroid gene expression pattern of the Wolf-Chaikoff effect supports the proposals that I_2_, as an oxidized form of iodine does not need TPO for its organification and that a putative iodine-containing organic compound that mediates these effects is generated [13]. These findings agree with previous data obtained in the MCF-7 cell line, where we found that ^125^I_2 _is taken up by a facilitated mechanism and that iodinated lipids with a migration similar to that of 6-IL are formed in a process that is not blocked by propylthiouracil (PTU) [14]. It is also interesting that 6-IL is present in I_2_-treated normal mammary glands, and the possibility that 6-IL at this concentration (0.13 nmol/g) could exert functional effects cannot be dismissed. An analysis investigating this is currently under progress.

Our present data show that I_2 _treatment significantly reduces PPARα and increases PPARγ expression, findings that could explain the antineoplasic effect of I_2_. It is well documented that in mammary cancer cells PPARα is over-expressed, and its activation correlates with proliferation [19,24]. In contrast, in the rat MNU-mammary cancer model dietary treatment with GW7845, an agonist of PPARγ, significantly reduced tumor incidence, tumor number, and tumor weight [25]. Moreover, the significant decrease in vasculature, as well as in VEGF and uPA expression observed in tumors from I_2_-treated rats, is additional evidence that PPAR receptors participate in the antineoplasic effect of I_2_. In fact, it has been reported that VEGF is upregulated by PPARα agonists [24,26], whereas uPA is inhibited by PPARγ activation [27]. VEGF is a potent mitogen with high specificity for endothelia, and it undergoes alternative splicing generating several isoforms [28]. We showed that isoforms 120 and 164, which are the most involved in angiogenesis, are present in MNU-induced tumors and that I_2 _treatment decreased the expression of both in a similar manner. The inhibition of PPARα expression might explain this effect, although more complex mechanisms could also be involved. Estrogen is known to induce VEGF expression [29], and recently it was reported that iodine inhibits the expression of several estrogen-responsive genes in MCF-7 cells [30]. Although the detailed mechanism involved in this effect has not been elucidated, PPARγ was shown to exert an inhibitory effect on estrogen-responsive genes via cross-talk with the estrogen receptor binding sites [31]. On the other hand, uPA is a 53-kDa trypsin-like protease that converts the zymogen plasminogen into active plasmin. Although uPA is a relatively specific protease, plasmin acts on a wide variety of protein substrates including metal proteases that destroy the extra cellular matrix (ECM), the degradation of which is a prerequisite for cancer invasion and metastasis [32]. uPA has been used as a specific marker for invasiveness in mammary cancer pathology. The presence of high concentrations of uPA in primary breast cancers correlates with a poor prognosis [33]. I_2_, after its conversion to 6-IL could, on the one hand, trigger the apoptotic cascades and on the other, decrease the invasive potential of cancer cells by inhibiting the expression of metastatic components like VEGF and uPA, which would account for the antineoplastic effect of I_2_.

In conclusion, these data support our notion that I_2 _supplement could be an adjuvant in the therapy of mammary cancer, where the high concentration of AA characteristic of tumoral cells serves as substrate to form 6-IL, which in turn triggers the activation of apoptotic and anti-invasive pathways by modulating PPAR receptors.

## Competing interests

The authors declare that they have no competing interests.

## Authors' contributions

CA: have made substantial contributions to conception and design; PGS: carried out some histological and molecular assays, OAH carried out some HPLC analysis, LVR carried out some histological and molecular assays, GD, carried out some HPLC analysis, BA carried out some molecular assay and analysis and interpretation of data. All authors have read and approved the final manuscript.
